# Role of the APOE polymorphism in carotid and lower limb revascularization: A prospective study from Southern Italy

**DOI:** 10.1371/journal.pone.0171055

**Published:** 2017-03-01

**Authors:** Sandra Mastroianno, Giuseppe Di Stolfo, Davide Seripa, Michele Antonio Pacilli, Giulia Paroni, Carlo Coli, Maria Urbano, Carmela d’Arienzo, Carolina Gravina, Domenico Rosario Potenza, Giovanni De Luca, Antonio Greco, Aldo Russo

**Affiliations:** 1 Cardiology Unit, Cardiological and Vascular Department, I.R.C.C.S. “Casa Sollievo della Sofferenza”, San Giovanni Rotondo, Foggia, Italy; 2 Complex Structure of Geriatrics, Medical Sciences Department, I.R.C.C.S. “Casa Sollievo della Sofferenza”, San Giovanni Rotondo, Foggia, Italy; Duke University, UNITED STATES

## Abstract

**Background:**

Atherosclerosis is a complex multifactorial disease and the apolipoprotein E (APOE) polymorphism has been associated to vascular complications of atherosclerosis.

**Objectives:**

To investigate the relationship between the APOE genotypes and advanced peripheral vascular disease.

**Materials and methods:**

258 consecutive patients (201 males and 57 females, mean age 70.83 ± 7.89 years) with severe PVD were enrolled in a 42-months longitudinal study (mean 31.65 ± 21.11 months) for major adverse cardiovascular events. At follow-up genotypes of the APOE polymorphism were investigated in blinded fashion.

**Results:**

As compared with ε3/ε3, in ε4-carriers a significant higher incidence of major adverse cardiovascular events (35.58% vs. 20.79%; p = 0.025) and total peripheral revascularization (22.64% vs. 5.06%; p < 0.001) was observed. Prospective analysis, showed that ε4-carriers have an increased hazard ratio for major adverse cardiovascular events (adjusted HR 1.829, 95% CI 1.017–3.287; p = 0.044) and total peripheral revascularization (adjusted HR = 5.916, 95% CI 2.405–14.554, p <0.001).

**Conclusions:**

The ε4 allele seems to be risk factor for major adverse cardiovascular events, and in particular for total peripheral revascularization in patients with advanced atherosclerotic vascular disease.

## Introduction

Atherosclerosis and associated cardiovascular diseases (CVD) represent the major cause of death and comorbidities in Western and developing countries [1, 2]. The Global Burden of Disease Study estimated that about 32% of all deaths worldwide in 2013 were caused by CVD, with about 80% of these deaths occurring in low-and middle-income countries [3]. In 2011, CVD still had a significant social burden in the US with annual costs for disease treating estimated about US$320.1 billion [4, 5]. Some manifestations of CVD as stroke, heart attack, limb ischemia, peripheral revascularization or amputation, have a decisive weight on health-related quality of life and disability, for which the long-term care costs tend to exceed health spending [6].

Atherosclerosis recognizes several risk factors, some determined by environment and modifiable, such as diet and smoking, other individual, and some non-modifiable, such as familiar CVD history, age and sex [7, 8]. Furthermore, the most common of these risk factors play a different role on the various arterial districts, such as low density lipoprotein-Cholesterol (LDL-Ch) for coronary heart disease, arterial systemic pressure for stroke, and smoking and systolic blood pressure for carotid and lower limbs disease [9, 10, 11]. In addition, genetic susceptibility in families affected by CVD is an important predisposing factor for atherosclerosis and for the most aggressive forms of vascular disease. In this regard, numerous studies have been conducted to identify susceptibility genes, which may have a predictive role in assessing the aggressiveness of the disease and the individual cardiovascular risk [12, 13]. Among these susceptibility genes, one of the most studied is the polymorphism of apolipoprotein E (APOE) gene [14, 15]. This polymorphism resulted in three most common alleles ε2, ε3, and ε4 and six most common genotypes [16, 17] that have been already related in different way to atherosclerosis and associated CVD [18, 19, 20]. Some epidemiological studies investigated the direct impact of APOE as a risk factor for coronary heart disease while others assessed the impact on cholesterol levels. APOE2 is mainly associated with lower LDL-Ch levels, while APOE4 allele with higher LDL-Ch levels [21, 22, 23]. Clinical studies have suggested a predisposing role for APOE4 in the development of CVD [24, 25, 26]. To date, there is disagreement concerning the APOE role on atherosclerosis involving the carotid and lower limb arteries [27, 28, 29]. Aim of the present study was to investigate in a cohort of patients affected by advanced atherosclerosis, a possible relationship between the APOE genotypes and the progression towards more aggressive forms of the disease, until requiring surgical or endovascular revascularization of peripheral arterial vessels.

## Material and methods

### Standard protocol approval, registration, and patient consent

This was a longitudinal study of a cardiovascular controlled case-series of patients fulfilling the Declaration of Helsinki [30], the guidelines for Good Clinical Practice [31], and the Strengthening the Reporting of Observational Studies in Epidemiology (STROBE) [32]. The approval of the study was obtained from the local Ethics Committees on human experimentation of "Casa Sollievo della Sofferenza" Hospital, represented by Cardinal Elio Sgreccia, Sister Elisa Cipollone, Dr. Rosa Giuseppa Frazzica, Dr. Giuseppe Fasanella, Professor Antonio Mangiacotti, Dr. Salvatore De Cosmo, Dr. Nicola Giuliani and Dr. Antonio Melchionda. Written informed consent for research was obtained from each patient.

### Patient’s recruitment

From November 1^st^, 2009 to November 30^th^, 2013 two hundred fifty eight patients (201 males and 57 females, mean age 70.83 ± 7.89 years, range from 45 to 88) consecutively attending the multidisciplinary atherosclerosis outpatient clinic of “Casa Sollievo della Sofferenza” Hospital were enrolled in the study.

### Inclusion/exclusion criteria

**Fig 1:**
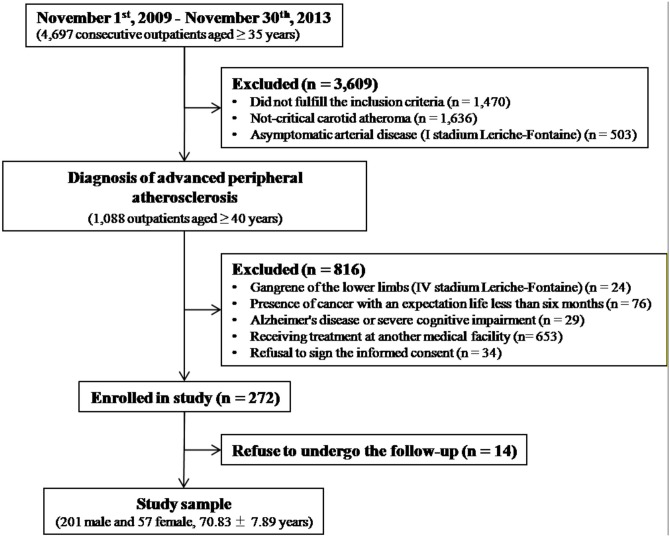
Summary of the study design.

Inclusion criteria were 1) Caucasian race, 2) written informed consent, and 3) a diagnosis of advanced atherosclerosis defined as carotid occlusion or severe stenosis (reduction of the vessel lumen greater than 50% evaluated with ultrasound) and/or lower limb ischemia II or III stadium Leriche-Fontaine (estimated by treadmill test at 12% inclination and 3.5 km/h speed) as already reported [33]. Exclusion criteria were presence of 1) not critical carotid atheroma, 2) I stadium Leriche-Fontaine (defined as asymptomatic arterial disease), 3) IV stadium Leriche-Fontaine (represented by gangrene of the lower limbs), 4) cancer with an expectation life less than six months, 5) clinical diagnosis of Alzheimer’s disease. A summary of the study design was reported in Fig 1.

### Clinical evaluation

Medical status was collected by a structured interview, a clinical evaluation, and a review of records from the patient’s general practitioners. In detail, patients underwent a clinical and laboratory examination for history of cancer, smoking habits, obesity, arterial hypertension, dyslipidemia, type 2 diabetes mellitus, as described in previously [33] and according to criteria of “The Third Report of The National Cholesterol Education Program” (NCEP ATP III) [34]. Lastly, serum concentrations of 25-hydroxy-vitamin D (25-OH-Vit D), erythrocyte sedimentation rate (ESR); homeostatic model assessment-insulin resistance (HOMAir); high sensitivity-C reactive protein (hs-CRP) and fibrinogen were assessed in all patients. Moreover, we assessed by strict analysis of clinical documentation the presence of previous CVD, defining as myocardial infarction, stroke, carotid or lower limb revascularization and myocardial reperfusion procedure.

At enrollment, arterial stiffness was evaluated by non-invasive method of brachial-ankle pulse wave velocity (PWV) and augmentation index using the AngE System (Sonotechnik, Austria, Europe), as already reported [33]. Standard 12-lead electrocardiogram and echocardiogram have been performed for determination of heart rhythms, PR, QRS, QTc intervals, bundle branch block, and for measure left ventricle ejection fraction (LVEF) and ventricular mass, respectively. The estimation of left ventricular mass index (LVMI) was performed dividing left ventricular mass to body surface. Doppler ultrasound examination was used for study course and size of the abdominal aorta, stenosis of carotid, vertebral, subclavian, renal, iliac, femoral, popliteal and tibial arteries. Renal resistance index was calculated at the renal hilum.

All the participants underwent a mean follow-up of 31.65 ± 21.11 months (range 6 to 50 months, mode 42 months). At follow up we have evaluated major adverse cardiovascular events (MACE), defined as myocardial infarction, cerebral ischemia, myocardial and/or peripheral revascularization and cardiovascular death (included all deaths for heart failure, myocardial infarction, stroke, malignant arrhythmias and sudden death). Furthermore, as additional data were recorded all deaths from malignant tumors.

### Genetic analysis

Genomic DNA was purified from whole blood by using standard methods [35]. The APOE genotypes were identified in blinded fashion as previously described [36]. The observed genotype frequencies were 10.47% for the ε2/ε3, 68.99% for ε3/ε3, 19.38% for ε3/ε4, and 1.16% for ε4/ε4. No ε2/ε2 or ε2/ε4 genotypes were identified. No differences were observed in respect to the expected Hardy-Weinberg frequencies (p = 0.622). According to these genotype frequencies, the estimated allele frequencies were 5.232 for the ε2 allele, 83.915 for ε3, and 10.853 for the ε4 allele. Agreeing to the APOE genotypes, patients were grouped as ε2 (ε2/ε2 + ε2/ε3), ε3 (ε3/ε3), and ε4 (ε4/ε3 + ε4/ε4) and the ε3 was used as the reference category (wild type).

### Statistical analysis

Continuous variables were reported as means ± standard deviation (SD) as well as categorical variables were reported as percentages. Dichotomous variables were compared by using the Pearson's χ^2^ test. Normal distribution of continuous variables was verified by the Kolmogorov-Smirnov test. Differences in the groups were analyzed using variance analysis (two-tailed unpaired *t*-test). Hardy-Weinberg equilibrium was tested by χ^2^test. *P* values <0.05 were considered statistically significant. Event-free survival in patients were calculated by Kaplan–Meier method and compared by log rank test. For the prospective analysis of cardiovascular events incidence in the groups Cox model were performed to estimate hazard ratio (HR) with 95% confidence interval (95% CI). HR was calculated with adjustment for the common risk factors as BMI, hypertension, diabetes, dislipidemia, smoking, age and gender. SPSS 13.0 software (Chicago, IL, USA) was used for statistical analysis.

## Results

Demographic and clinical characteristics of patients at baseline according to the APOE genotypes were reported in Table 1. In respect to ε3, we found ε2-patients significantly more obese (BMI 29.81 ± 2.52 Kg/m^2^ vs. 28.63 ± 4.13 Kg/m^2^; p = 0.046), have lower levels of 25-OH-Vit D (10.78 ± 6.45 ng/ml vs. 17.02 ± 14.08 ng/ml; p = 0.001), greater arterial stiffness as PWV (17.00 ± 3.60 m/sec vs. 14.45 ± 4.17 m/sec; p = 0.001), and an increased LVMI (89.69 ± 25.35 gr/m^2^ vs. 76.52 ± 19.80 gr/m^2^; p = 0.017). Statistical trend towards a greater waist-hip ratio (0.99 ± 0.06 vs. 0.96 ± 0.06; p = 0.053) and a lower level of LDL-Ch (81.80 ± 48.52 mg/dl vs. 96.28 ± 33.21 mg/dl; p = 0.050) were also observed. Even in respect to ε3, we found ε4-patients significantly less obese (BMI 27.23 ± 3.63 Kg/m^2^ vs. 28.63 ±4.13 Kg/m^2^; p = 0.029), and with minor inflammatory indexes as ESR (21.21 ± 13.02 mm/h vs. 26.66 ± 18.52 mm/h; p = 0.026) and hs-CRP (0.42 ± 0.35 mg/dl vs. 0.80 ± 2.69 mg/dl; p = 0.016). Statistical trend towards a minor level of fibrinogen (327.98 ± 68.37 mg/dl vs. 352.26 ± 78.68 mg/dl; p = 0.054) was also observed.

**Table 1:** Demographic and clinical characteristics of patients at baseline according to APOE genotype.

	APOE genotypes			All
	ε2/ε2 + ε2/ε3	ε3/ε4 + ε4/ε4	ε3/ε3	
Number of subjects	27	53	178	258
Age (years)	71.26 ± 7.89	70.55 ± 8.32	70.84 ± 7.81	70.83 ± 7.89
Gender (male/female)	20/7	42/11	139/39	201/57
BMI (kg/m^2^)^a^^,^^b^	29.81 ± 2.52	27.23 ± 3.63	28.63 ± 4.13	28.47 ± 3.94
Waist circumference (cm)	102.32 ± 8.79	98.80 ± 13.54	101.26 ± 9.85	100.86 ± 10.63
Waist-hip ratio	0.99 ± 0.06	0.96 ± 0.10	0.96 ± 0.06	0.97 ± 0.07
Systolic blood pressure (mmHg)	133.24 ± 15.93	134.52 ± 17.11	132.69 ± 17.76	133.12 ± 17.31
Diastolic blood pressure (mmHg)	80.65 ± 7.06	80.00 ± 6.42	79.16 ± 6.23	79.48 ± 6.36
Pulse pressure (mmHg)	52.59 ± 13.01	54.52 ± 14.92	53.53 ± 15.76	53.63 ± 15.32
Fasting glucose (mg/dl)	123.70 ± 46.89	118.85 ± 42.57	117.37 ± 36.93	118.34 ± 39.14
HOMAir	8.92 ± 24.36	4.07 ± 5.47	5.02 ± 8.09	5.25 ± 10.72
Triglycerides (mg/dl)	136.44 ± 75.97	110.29 ± 41.41	198.53 ± 55.71	111.75 ± 55.87
Total Ch (mg/dl)	156.81 ± 53.13	165.92 ± 37.34	168.09 ± 39.58	166.45 ± 40.75
HDL-Ch (mg/dl)	47.74 ± 11.70	49.56 ± 12.21	48.45 ± 12.53	48.60 ± 12.35
LDL-Ch (mg/dl)	81.80 ± 48.52	94.41 ± 35.04	96.28 ± 33.21	94.36 ± 35.59
Serum uric acid (mg/dl)	5.50 ± 1.44	5.40 ± 1.51	5.47 ± 1.37	5.46 ± 1.41
25-OH-Vit D (ng/ml)^c^	10.78 ± 6.45	13.73 ± 9.01	17.02 ± 14.08	15.68 ± 12.72
Fibrinogen (mg/dl)	339.96 ± 72.79	327.98 ± 68.37	352.26 ± 78.68	346.15 ± 76.48
ESR (mm/h)^d^	24.64 ± 15.72	21.21 ± 13.02	26.66 ± 18.52	25.33 ± 17.34
hs-CRP (mg/dl)^e^	0.61 ± 0.66	0.42 ± 0.35	0.80 ± 2.69	0.70 ± 2.27
Serum creatine (mg/dl)	1.01 ± 0.32	0.99 ± 0.30	1.04 ± 0.63	1.03 ± 0.55
eGFR (ml/min/1.73 m^2^)	74.97 ± 19.34	80.92 ± 22.56	82.15 ± 28.53	81.14 ± 26.56
Microalbuminuria (μg/min)	76.11 ± 144.11	77.92 ± 182.92	77.05 ± 183.86	77.13 ± 179.03
Renal resistance index	0.69 ± 0.06	0.68 ± 0.07	0.68 ± 0.06	0.68 ± 0.06
ABI	0.90 ± 0.12	0.92 ± 0.17	0.93 ± 0.12	0.93 ± 0.13
Augmentation index (%)	22.78 ± 5.02	21.63 ± 8.36	23.16 ± 8.03	22.82 ± 7.84
PWV (m/sec)^f^	17.70 ± 3.60	13.32 ± 5.88	14.45 ± 4.17	14.56 ± 4.63
PR interval	172.64 ± 27.53	175.78 ± 58.08	164.56 ± 27.08	167.66 ± 35.84
QRS interval	99.44 ± 23.80	104.17 ± 19.93	101.62 ± 22.48	101.90 ± 22.09
QTc interval	413.64 ± 25.16	408.74 ± 52.50	409.79 ± 42.99	409.99 ± 43.46
Heart rate (bpm)	71.81 ± 9.63	68.16 ± 11.29	70.36 ± 10.66	70.06 ± 10.70
LVEF (%)	58.54 ± 4.97	57.55 ± 6.74	58.112 ± 5.96	58.05 ± 6.01
LVMI (gr/m^2^)^g^	89.69 ± 25.35	75.03 ± 20.60	76.52 ± 19.80	77.70 ± 21.00

ε2/ε2 + ε2/ε3 vs. ε3/ε3:

^a^p = 0.046;

^c^p = 0.001;

^f^p = 0.001;

^g^p = 0.017.

ε3/ε4 + ε4/ε4 vs. ε3/ε3:

^b^p = 0.029;

^d^p = 0.026;

^e^p = 0.013.

Data are presented as mean ± SD. 25-OH-Vit D: 25-hydroxy-vitamin D; ABI: Ankle brachial index; BMI: Body mass index; eGFR: Estimated glomerular filtration rate; ESR: Erythrocyte sedimentation rate; HDL-Ch: High density lipoprotein—cholesterol; HOMAir: Homeostatic model assessment—insulin resistance; hs-CRP: High sensitivity—C reactive protein; LDL-Ch: Low density lipoprotein—cholesterol; LVEF: Left ventricular ejection fraction; LVMI: Left ventricular mass index; PWV: Pulse wave velocity.

Overall, no differences were observed between comorbidities, smoking habits and concomitant drug treatments of patients at baseline, as reported in Table 2.

**Table 2:** Comorbidities and medical treatments at baseline according to APOE genotypes.

	APOE genotypes						All	
	ε2/ε2 + ε2/ε3		ε3/ε4 + ε4/ε4		ε3/ε3			
**Comorbidities**								
Hypertension	24	(88.89%)	51	(96.23%)	163	(91.57%)	238	(92.25%)
Dyslipidemia	25	(92.59%)	49	(92.45%)	168	(94.38%)	242	(93.80%)
Type 2 diabetes	15	(55.55%)	29	(54.72%)	92	(51.68%)	136	(52.71%)
Smoking	7	(25.92%)	10	(18.87%)	46	(25.84%)	63	(24.42%)
Myocardial infarction	5	(18.51%)	7	(13.21%)	33	(18.54%)	45	(17.44%)
Stroke	3	(11.11%)	7	(13.21%)	22	(13.36%)	32	(12.40%)
Carotid revascularization	8	(29.63%)	11	(20.75%)	38	(21.35%)	57	(22.09%)
Lower limb revascularization	2	(7.41%)	10	(18.87%)	27	(15.17%)	39	(15.12%)
Myocardial revascularization	8	(29.63%)	20	(37.73%)	46	(25.84%)	74	(28.68%)
Cancer	5	(18.52%)	10	(18.87%)	48	(26.97%)	63	(24.42%)
**Medical treatments**								
ARBs	9	(33.33%)	25	(47.17%)	68	(38.20%)	102	(39.53%)
ACE inhibitors	9	(33.33%)	18	(33.96%)	71	(39.88%)	98	(37.89%)
Calcium channel blockers	11	(40.74%)	18	(33.96%)	48	(26.96%)	77	(29.84%)
β-blockers	4	(14.81%)	16	(30.19%)	42	(23.59%)	62	(24.03%)
Diuretics	15	(55.55%)	23	(43.40%)	73	(41.01%)	111	(43.02%)
Antiplatelet	22	(41.48%)	42	(79.25%)	157	(88.20%)	221	(85.66%)
Statin	24	(88.88%)	42	(79.25%)	158	(88.76%)	224	(86.82%)
Allopurinol and/or febuxostat	1	(3.70%)	3	(5.66%)	20	(11.23%)	24	(9.30%)
Antidiabetic therapy								
Diet	6	(40.00%)	7	(24.14%)	25	(27.17%)	38	(14.73%)
Oral hypoglycemic	8	(53.33%)	10	(34.48%)	47	(51.08%)	65	(25.16%)
Insulin + Oral hypoglycemic	1	(6.66%)	12	(41.38%)	20	(21.73%)	33	(12.79%)

Data are presented as number (%) of subjects. ACE inhibitors: angiotensin converting enzyme inhibitors; ARBs: angiotensin receptor blockers.

Cardiovascular adverse events at follow-up according to APOE genotypes are presented in Table 3. As compared to ε3, in ε4-patients a significant higher incidence of MACE (35.58% vs. 20.79%; p = 0.025), as well as carotid (15.09% vs. 5.06%; p = 0.014) and lower limb (13.21% vs. 1.69%; p < 0.001) revascularizations were observed. Accordingly, an higher incidence of total peripheral revascularizations (defined as carotid plus lower limb revascularizations) in ε4 was observed (22.64% vs. 5.06%; p < 0.001). Notably, an increased cancer-related mortality in ε2-carriers was also observed respect to ε3 (11.11% vs. 2.25%; p = 0.018).

**Table 3:** Events at follow-up.

	APOE genotypes						All	
	ε2/ε2 + ε2/ε3		ε3/ε4 + ε4/ε4		ε3/ε3			
**Cardiovascular events**								
MACE^a^	5	(18.52%)	19	(35.85%)	37	(20.79%)	61	(23.64%)
Acute	-	-	5	(9.43%)	16	(8.99%)	21	(8.14%)
**Revascularizations**								
Myocardial	-	-	1	(1.89%)	14	(7.8/%)	15	(5.81%)
Carotid^b^	3	(11.11%)	8	(15.09%)	9	(5.06%)	20	(7.75%)
Lower limb^c^	-	-	7	(13.21%)	3	(1.69%)	10	(3.88%)
Total peripheral ^d^	3	(11.11%)	12	(22.64%)	9	(5.06%)	24	(9.30%)
**Fatal events**								
Total death	3	(11.11%)	6	(3.37%)	10	(5.61%)	19	(7.36%)
Cardiac death	-	-	4	(7.55%)	6	(3.37%)	10	(3.88%)
Cancer death^e^	3	(11.11%)	2	(3.77%)	4	(2.25%)	9	(3.49%)

ε3/ε4 + ε4/ε4 vs. ε3/ ε3:

^a^p = 0.025;

^b^p = 0.014;

^c^p < 0.001;

^d^p < 0.001

ε2/ ε2 + ε2/ε3 vs. ε3/ε3:

^e^p = 0.018.

Data are presented as number (%) of subjects.

MACE: Major adverse cardiac events.

**Fig 2:**
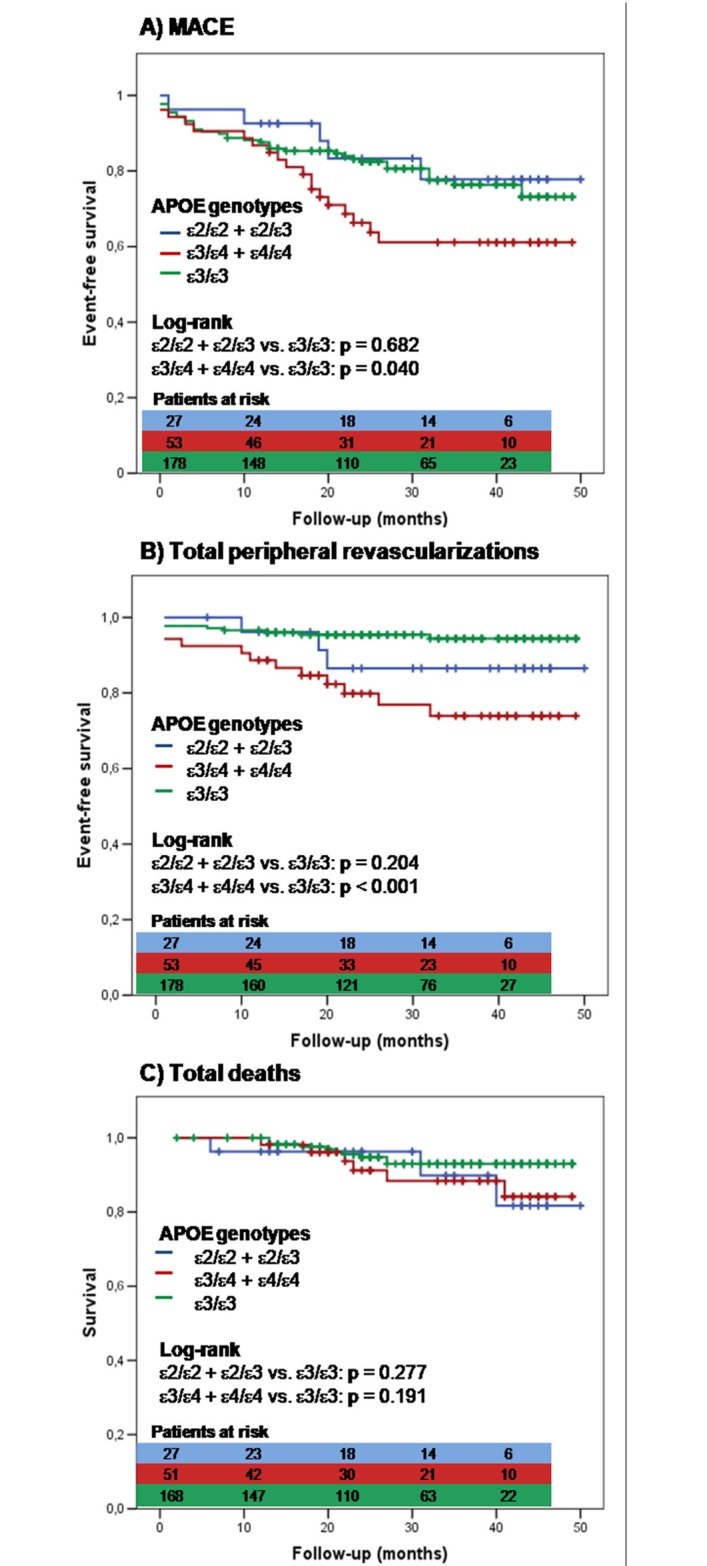
Kaplan–Meier estimates of the adverse events according to genotype. A) Major adverse cardiac events (MACE), B) Total peripheral revascularizations, C) Total deaths.

Events-free survivals associated to the APOE status were presented in Fig 2. As compared with ε3, ε4-patients showed a significantly lower MACE-free survival (p = 0.040, Fig 2A), and lower total peripheral revascularizations-free survival (p < 0.001, Fig 2B). No statistically significant difference was observed between free survival curves from total deaths (Fig 2C).

Prospective analysis (Table 4), showed that ε4-carriers, in respect to ε3, have a higher risk of MACE (adjusted HR 1.829, 95% CI 1.017–3.287; p = 0.044), and total peripheral revascularizations (adjusted HR = 5.916, 95% CI 2.405–14.554; p < 0.001) as resulted from the single revascularization item of carotid (adjusted HR = 3.550, 95% CI 1.291–9.765; p = 0.014) and lower limb (adjusted HR = 9.607, 95% CI 2.405–14.554; p = 0.002).

**Table 4:** Hazard ratios for incident cardiovascular events.

	ε2/ε2 + ε2/ε3 vs. ε3/ε3				ε3/ε4 + ε4/ε4 vs. ε3/ε3			
	Crude estimated		Adjusted estimates		Crude estimated		Adjusted estimates	
	HR	p	HR	p	HR	p	HR	p
	(95% CI)		(95% CI)		(95% CI)		(95% CI)	
**Cardiovascular events**								
MACE	1.214	0.684	1.134	0.796	1.766	0.044	1.829	0.044
	(0.476–3.095)		(0.438–2.937)		(1.015–3.073)		(1.017–3.287)	
Acute	-	-	-	-	1.021	0.968	1.347	0.578
					(0.374–2.787)		(0.471–3.852)	
**Revascularizations**								
Myocardial	-	-	-	-	0.226	0.151	0.283	0.226
					(0.030–1.718)		(0.037–2.189)	
Carotid	0.447	0.228	0.437	0.229	3.066	0.021	3.550	0.014
	(0.121–1.652)		(0.113–1.684)		(1.183–7.947)		(1.291–9.765)	
Lower limb	-	-	-	-	8.022	0.003	9.607	0.002
					(2.074–31.031)		(2.357–39.159)	
Total Peripheral	0.439	0.218	0.494	0.310	4.746	<0.001	5.916	<0.001
	(0.119–1.624)		(0.127–1.927)		(1.999–11.268)		(2.405–14.554)	
**Fatal events**								
Total deaths	0.496	0.288	0.394	0.191	1.939	0.200	1.155	0.806
	(0.136–1.805)		(0.098–1.590)		(0.704–5.341)		(0.366–3.650)	
Cardiac death	-	-	-	-	2.115	0.247	0.765	0.756
					(0.595–7.512)		(0.141–4.147)	
Cancer death	0.206	0.039	0.260	0.084	1.667	0.555	1.486	0.656
	(0.046–0.922)		(0.057–1.196)		(0.305–9.101)		(0.260–8.504)	

MACE: Major adverse cardiac events.

## Discussion

The present study analyzed the association of the APOE polymorphism with the incidence of cardiovascular events and death occurred in a short-term follow-up study of a cohort of 258 patients affected by advanced atherosclerosis. Our results showed that the ε4 allele seems to be risk factor for MACE, and in particular for total peripheral revascularizations in these patients.

Notably, our study focuses on a very-high risk elderly population, affected by known peripheral atherosclerosis with large prevalence of previous major cardiovascular events (stroke, myocardial infarct, peripheral and cardiac revascularization) and presenting traditional risk factors already managed by a tailored treatment approach. Most part of previous studies analyzed several genes in population affected by mild atherosclerosis, thus enrolling patients at not very-high cardiovascular risk [25, 37].

The other part of these studies are represented by large epidemiological studies with medium-term follow up [38]. More recently, the SMART study analyzed APOE relation with PVD in a wide sample of patients aged 56.7 ± 12.4 years (72% of subjects with manifest CVD and 28% with only risk factors), showing incidence of more cardiovascular events and peripheral revascularizations in ε2/ε2 subgroup; otherwise, this relation was partially explained by different non-HDL-Ch and inhomogeneity in previous events, not present in our population [28].

The present study is a small, single-center study with a short follow-up. Nevertheless the population is homogeneous and well defined for clinical and therapeutic characteristic. Accordingly, in this condition we have found an increased incidence of MACE in ε4-carriers, and in particular a significant incidence of lower limb and carotid revascularizations in this group. The present study finds no association between the polymorphism and all-cause mortality as a previous study on Italian diabetic patients [39].

Although some studies have shown increased risk of myocardial revascularization and adverse events after coronary artery bypass in ε4-carriers [19, 40], in our population we did not replicate this finding; at least a selection bias could be represented by abovementioned selection criteria.

In the evaluated population, we found a significant increase of cancer mortality in the ε2-patients; this finding has not been declared as the object of the study; nevertheless, this result, partially corroborated by scientific literature [41, 42], yet still controversial at some meta-analysis [43, 44], remains of interest for further studies. Our study follows an observational prospective approach, therefore we can only infer association link, more than causal relationship; however this limit is common to many previous researches. A longer follow up and the enlargement of patient number could provide a better understanding on the role of APOE in patients affected by advanced atherosclerosis.

In several multiethnic studies, APOE polymorphism did not have a strong correlation with BMI [12, 45]; once again our study, according to research on ethnically homogeneous populations in Croatia [46], showed higher BMI in ε2-carriers compared to reference group. Furthermore, we found lower BMI in ε4-carriers. These differences, within ethnically homogeneous populations, could be explained by the action of adjacent genes, segregating in linkage disequilibrium with APOE allele [47, 48].

Previous study of Huebbe et al. shows higher vitamin D levels in mice and humans ε4-carriers [49]; otherwise our data show significant lower level 25-OH-Vit D in ε2-patients. Since lower level of 25-OH-Vit D are associated to artery calcification [50], this could explain the relationship between dissimilar calcified atherosclerosis in ε2, ε4 and ε3-carriers. Of great interest, a different at atherosclerosis process based on various grade of atheroma and wall vessels calcification could determine a plaque stabilization, as suggested by Nandalur [51], inducing a slower disease progression in ε2 than in ε4-carriers.

Evaluation of PWV showed that ε2-carriers have greater arterial stiffness, expression of vessel calcifications burden [52]. Instead of the study presented by Alvim [53], our results could be determined by ethnic homogeneity of our population; at same time, in our cohort, the PWV shows a correlation with ventricular mass indexed in ε2-patients, expression of organ damage, yet the evaluation of arterial stiffness was not able to stratify the cardiovascular risk in the setting of advanced atherosclerosis during follow-up (data not showed).

Analysis of lipid levels in enrolled patients has not showed significant differences regarding to the APOE polymorphism, as already receiving optimal statin treatment for secondary prevention. The known complex association between apolipoprotein E and lipid levels justifies the genetically determined risk of developing CVD. Moreover, numerous scientific evidence showed that apolipoprotein E plays a pleiotropic effect beyond dyslipidemia; in particular, at the level of atherosclerotic plaque the expression of apolipoprotein E by macrophages might have an important role in the determination of plaque instability not only attributable to the progression of the degree of stenosis [54, 55].

Literature showed that atherosclerosis is an inflammatory disease within the arterial wall, responsible for several important events such as coronary and peripheral vascular disease [56]. In the studied population, ESR and hs-CRP do not correlate with aggressiveness of disease observed in ε4-carriers. It could be due to patient’s characteristics, affected by already severe atherosclerosis at enrollment, or to an independent and important role played by the gene.

In our population, APOE4 provide further cardiovascular prognostic stratification; this finding implies a huge role of APOE polymorphism for better shaping individual additional residual risk stratification. Although recent analysis are not conclusive in cardiovascular event prediction by using genetic risk score [38], in the future, this could lead to an improved clinical decision making by more aggressive treatment of those subjects at greatest genetic risk, economizing treatment for those at less risk, and therefore translating pharmacogenetics and individualized medicine in finer health system cost-effectiveness [57].

## Conclusions

In our cohort of patients at very high-risk of cardiovascular events the APOE4 may be used for a further prognostic stratification for MACE and peripheral revascularization, adding more information useful for individual residual risk determination. Prospectively, this finding would improve pharmacogenetics, leading to the "best marriage" between individualized medicine and health system cost-effectiveness. Clearly APOE polymorphisms represent just a brick in the Great Wall of atherosclerosis building, yet still having a definite weight in the physiopathology of this complex disease.

## Supporting information

S1 File Dataset. (XLS)

## Abbreviations

25-OH-Vit D: 25-hydroxy-vitamin D
ACE inhibitors: Angiotensin Converting Enzyme Inhibitors
APOE: apolipoprotein E encoding gene
ARBs: Angiotensin Receptor Blockers
BMI: Body Mass Index
CI: Confidence Interval
CVD: Cardiovascular Disease
eGFR: Estimated Glomerular Filtration Rate
ESR: Erythrocyte Sedimentation Rate
HDL-Ch: High Density Lipoprotein-Cholesterol
HOMAir: Homeostatic Model Assessment—Insulin Resistance
hs-CRP: High Sensitivity—C Reactive Protein
HR: Hazard Ratio
LDL-Ch: Low Density Lipoprotein–Cholesterol
LVEF: Left Ventricle Ejection Fraction
LVMI: Left Ventricular Mass Index
MACE: Major Adverse Cardiovascular Events
NCEP ATP III: Third Report of The National Cholesterol Education Program
PVD: Peripheral Vascular Disease
PWV: Pulse Wave Velocity
SD: Standard Deviation
